# Analysis of Issues and Future Trends Impacting Drug Safety in South Korea

**DOI:** 10.3390/ijerph16183368

**Published:** 2019-09-12

**Authors:** Myeong Gyu Kim, Seungyeon Huh, Nayoung Han, Jae Hyun Kim, Kyungim Kim, Euni Lee, In-Wha Kim, Jung Mi Oh

**Affiliations:** 1 Graduate School of Clinical Pharmacy, CHA University, 11160 Pocheon, Korea; 2 College of Pharmacy and Research Institute of Pharmaceutical Sciences, Seoul National University, 08826 Seoul, Korea; 3 College of Pharmacy, Korea University, 30019 Sejong, Korea

**Keywords:** drug safety, safety management, future, trend, forecasting

## Abstract

New drug safety issues are emerging that are beyond the existing medication safety management system. To pre-empt these problems, forecasting future drug safety trends and issues is a necessity. The objective of this study was to identify issues and future trends impacting drug safety using foresight methodologies. The study started by identifying global megatrends, trends in safety management of medicines, and key issues in drug safety. A total of 25 global megatrends were selected by extracting and clustering keywords from 26 reports concerning the future. Using the text-mining method, 10 trends in drug safety were identified from 3593 news articles. This study derived 60 issues which can arise from the trends, and finally, the 20 key issues with the highest urgency and impact scores were selected. Some examples of issues with high scores were as follows: illegal distribution of medicines, lack of technology for managing and utilizing big data, change in the pharmaceutical trade environment, lack of education and safety management for specific populations, lack of artificial intelligence-based technology for the safety management of medicines, and the prevalence of drug advertisements through social network services. The key issues could be used to establish plans for medication safety management.

## 1. Introduction

Because a medication safety accident can cause harm to patients or death in severe cases, it is a threat to patient safety. The recent recall of antihypertension medicines due to carcinogenic substances contained in the China-sourced raw materials caused great social problems. Moreover, the number of cases in which medication is recalled or suspended in the USA and South Korea is estimated to be around 40 cases per year in both countries [1,2]. Because of the continuous occurrence of medication safety accidents, the public’s demand for the more thorough safety management of medications is increasing. Currently, medication safety management is carried out with premarket scientific evaluations, postmarket re-evaluations, and the reporting of adverse drug reactions [3].

The Ministry of Food and Drug Safety (MFDS), like the USA Food and Drug Administration (FDA), regulates medical products in South Korea and conducts research and development (R&D) on a medication safety management. The project includes the advancement of policies and systems, scientific reviews and assessments, and guidelines for the safe use of medical products [4]. The MFDS publishes a white paper every year highlighting their achievement and describing the implementation plan for drug safety. White papers published in 2016 and 2017 emphasized (1) the introduction and stabilization of good manufacturing practices (GMP) that are in harmony with international standards; (2) the internationalization of medicine approval and an evaluation system; (3) strengthening safety management of approved pharmaceuticals; (4) strengthening the competitiveness of the pharmaceutical industry by stable operation in the patent-regulatory approval linkage system; (5) the establishment of a management system for preventing abuse and misuse of narcotic drugs [5,6].

As the National Assembly has pointed out, issues such as the distribution of medicines through social network services, the theft and loss of 186 opioid drugs, and the lack of management of the nation’s essential drugs, show that there are holes in medication safety management [7]. Furthermore, as modern society changes rapidly, a new safety issue may arise that is beyond the existing medication safety management system. The recent issue of carcinogenic Chinese raw materials in South Korea medication is the dark side of the globalization of pharmaceutical production and distribution [8]. Moreover, emerging social issues such as the aging society and low birth rates [9], the emergence of a new technological paradigm in medication quality management [10], and the emergence of new medicines beyond the conventional concept of medicine could have a negative impact on medication safety management [11]. With the rapid development of technology and the interrelation of technology and society, the future of society is likely to become more complex and uncertain, and the need for proactive preparation to address potential threats to future drug safety is essential. In light of these situations, the Act on the Promotion of Technology for Ensuring the Safety of Food, Drugs, etc. was enacted in 2015 and the Act states that a plan for the promotion of safety technologies should be established every five years [12]. Thus, the MFDS conducted a planning study to find R&D tasks for future medication safety management. This paper is part of the planning study.

The purpose of this study was to analyze the issues and future trends impacting drug safety using scientific foresight methodologies to timely respond to the rapidly changing global environments.

## 2. Materials and Methods

The definition of terms used in this study are as follows [13,14]: (1) global megatrends: a set of changes in society, technology, economy, environment, and political conditions which effects are not restricted to a particular geographic area; (2) trends in drug safety: a pattern of gradual change in the area of drug safety toward the future; (3) issues: problems or concerns that are expected to arise in the future; (4) key issues: issues that have great potential to affect our society.

**Figure 1:**
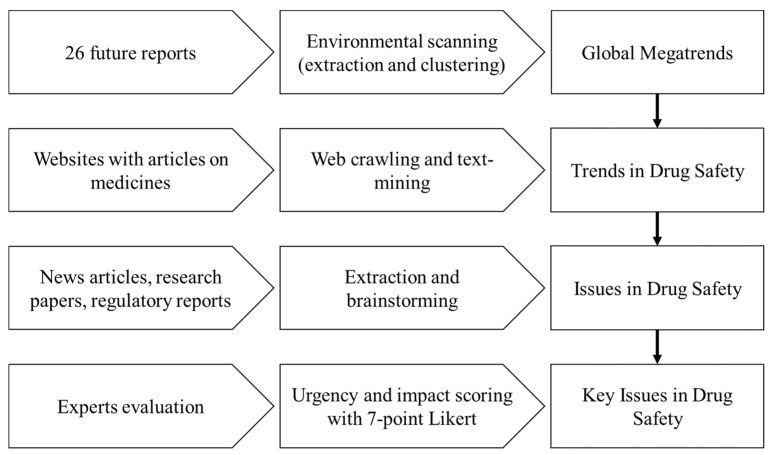
The flow of finding issues in drug safety.

Global megatrends, trends in drug safety, and key issues in drug safety were identified using a method referring to foresight methodologies used by Ministry of Science, Information and Communication Technology (ICT) and Future Planning, and Korea Institute of Science and Technology Evaluation and Planning (MSIP and KISTEP; Figure 1) [13]. We modified the method to suit the scope of drug safety. The scope of the drug safety considers the whole life cycle of drugs, which runs from premarket to postmarket, and it covers the advancement of policies, scientific reviews and assessments, quality evaluation of medical products, and the safe use of medical products according to the MFDS notice [15]. Biologics and herbal medicines were beyond the scope of the study.

Global megatrends were derived using the environmental scanning methodology [16]. First, twenty-six reports concerned with the future published since 2010 were selected as sources of data and are listed in Table S1 [17,18,19,20,21,22,23,24,25,26,27,28,29,30,31,32,33,34,35,36,37,38,39,40,41,42]. All trend keywords were extracted from the sources and then were categorized into STEEP (Social, Technological, Economic, Environmental, and Political). The classified keywords were clustered in several groups based on similarity and were named as global megatrends.

In order to derive trends in drug safety, text-mining was conducted. Unlike qualitative conventional methods such as Delphi, expert panels, and scenarios, which rely on opinions from experts, text-mining can forecast the future in objective and quantitative ways [43,44]. In addition, text-mining can save money and time when deriving trends as compared to costly and time-consuming literature reviews and experts’ advice [43,44]. A database of news websites about medicines in South Korea (http://www.yakup.com) was used. Other sources for text-mining were not used to limit overestimating problems due to overlapping articles on the same topic. As a search term, the terms corresponding to the global megatrend and the MFDS’s drug safety categories were used. A web crawler, using Python version 3.4 (Python Software Foundation, Delaware, USA), automatically collected and stored the body of the articles which were the <div class = “bodyarea”> part of the pages published from 1 January 2014, to 28 February 2017. Afterward, the words corresponding to the nouns were extracted by conducting an impropriety, and a stem extraction and morpheme analysis from the article text were collected using the R-program’s KoNLP (Heewon Jeon, South Korea) package. The simultaneous occurrence probability among the nouns was calculated using the latent Dirichlet allocation (LDA). The words that appear together are grouped into a topic. The trends in drug safety were selected to represent the nouns included in each topic. Since the search terms used for text-mining are the term determined by the megatrends, we could pair megatrends to trends, which were the results of text-mining.

Issues that may arise within five years concerning the medication safety trend were derived from a literature review and brainstorming. News articles, research papers, and regulatory agency reports from the USA, European Union, and Japan about trends in drug safety were reviewed. After that, both individual and group brainstorming sessions were conducted. Ten participants with one to more than ten years of experience in medicine performed brainstorming individually, and three of them participated in group brainstorming. Created issues were categorized. The issues were evaluated by thirteen experts from industries, universities, and research institutes assessing the urgency and impact using a 7-point Likert scale. Experts were evenly selected for industry, academia, and research institutes who had more than 20 years of experience and competence in planning research with sufficient insight into medication safety management. Urgency was defined as how quickly an issue will it be a problem or will need to be resolved. Impact was defined as how much the problem threatened people’s health or how much risk could be prevented when the problem was solved. Issues with a minimum average score of 4 (neutral) were chosen as key issues in drug safety, and issues that experts disagreed (an average score of less than 4) with were excluded.

## 3. Results

### 3.1. Global Megatrends and Trends in Drug Safety

A total of 517 trend keywords were extracted from 26 sources, and 25 global megatrends were derived by clustering the keywords. The 25 global megatrends are listed in Table S2. By STEEP, there were seven social trends, seven technological trends, two environmental trends, five economic trends, and four political trends.

The existing future reports did not describe trends for the field of drug safety, so trends in drug safety were derived using a website where medication-related news articles are posted. A total of 3593 articles related to both global megatrends and drug safety were extracted. Words extracted through text-mining were grouped into ten topics according to their probability of appearing simultaneously. Ten trends in drug safety were selected and are shown in Table 1.

### 3.2. Key Issues in Drug Safety

We obtained 60 issues in the drug safety area through literature review or brainstorming. The issues derived were eventually grouped into 24 issues. The twenty-four issues and their urgency and impact scores are presented in Table 2. The average score was 4.47. Twenty key issues with an average score of more than four were identified. ‘Illegal distribution of medicines’ got the highest score of 5.52 points followed by ‘Lack of technology for managing and utilizing big data’, ‘Change in the pharmaceutical trade environment’, ‘Lack of education and safety management for specific populations (pediatrics, elderly, etc.)’, ‘Lack of artificial intelligence-based technology for safety management of medicines’, and ‘Prevalence of drug advertisements through social network services’.

## 4. Discussion

We derived 10 trends and 20 key issues in drug safety. Foresight methodologies, which were environmental scanning and text-mining, were used in the research process. It is the first time in South Korea that text-mining has been used to forecast the future of drug safety. Planning research for medication safety management has been conducted previously. Science and Technology Policy Institute (STEPI) reports from 2001 documented safety management trends, such as the rapid development of the biological industry and the government’s will to foster it, increased investment by large companies and venture startups, the emergence of new biotechnology products, and the increase of new harmful and toxic substances [45]. It proposed implementing R&D projects for the safety assessment of technologies, establishing an international level of research infrastructure, strengthening the organization’s corporate support activities, introducing advanced systems for improving the service of businesses and the public, and operating a national toxic substances management program [45]. In 2010, researchers in South Korea forecasted the aging population and subsequent changes in disease structure, increasing medical costs, the pursuit of quality of life, the need for information, the opening of the market through free trade agreements, globalization of technological development, and the development of science and technology [46]. Furthermore, they suggested mid-long term strategies for the advancement of the drug safety system and the competitiveness of the pharmaceutical industry in South Korea based on the forecasting [46]. Some of their forecasts were realized in the Precision Medicine Initiative and the Sentinel Initiative, which are examples of active drug monitoring using big data in the United States [47]. On the other hand, the introduction of artificial intelligence, IBM’s Watson, USA, into medical care and expectations for the unification of the Korean peninsula following the election of a new president in South Korea will present new impacts on the safety of medicines. Therefore, future foresight research in drug safety is necessary periodically to respond to changing circumstances and to make plans.

The social, technological, economic, environmental, and political megatrends, such as the aging of society, advanced technologies, disasters, major countries’ low growth, and the unification of Korea affect drug safety. Unification between South and North Korea is a unique situation. North Korea has a significant gap in medicine safety management as compared to more advanced South Korea, and the terminology in drug management and disease prevalence are also different. Thus, efforts and studies are needed to narrow this gap [48]. Some trends were derived from only one megatrend search term, while others were derived from more than one megatrend. For example, ‘Drug safety for the aged, pregnant women and multicultural families’ trend is associated with megatrends of ‘Structural and functional changes of social members’ and ‘Globalization’. However global megatrends which were related with advanced technology such as ‘Cognitive science’ and ‘Space engineering’ were not relevant to trends in drug safety because there were no intersections between these technologies and medical products. In this study, trends were derived by text-mining, and it has been reported that there is no significant difference between the result from qualitative methods by experts and that of text-mining [43].

Issues related to the recent development of information technology were particularly high in urgency and impact. This reflects the high interest in managing drug safety by applying new big data and artificial intelligence technologies. Advertising or distributing medicines over the Internet is also a new problem. The key issues in drug safety can be used to determine the direction of policy proposals or the direction of R&D by MFDS to proactively address the drug safety issues forecasted by future environmental changes [49,50]. For example, key issues such as ‘Illegal distribution of medicines’ and ‘Change in the pharmaceutical trade environment’ are issues that need policy solutions. On the other hand, key issues such as ‘Lack of technology for managing and utilizing big data’ and the ‘Lack of artificial intelligence-based technology for safety management of medicines’ are issues that require R&D to address the drug safety issues.

This research has derived trends and issues in drug safety using scientific foresight methodologies but has several limitations. First, because trends are chosen based on words that have emerged with a high frequency in text-mining, it is likely that emerging trends, which are currently not discussed in articles, but maybe major in the future could be omitted. Second, some issues, such as unification, are unique to Korea and thus difficult to generalize when considering other countries. However, most of the issues are relevant to global society, so are worth mentioning.

Although accurate forecasting of the future of drug safety is not possible, research using foresight methodologies can proactively develop response strategies by presenting the possible future of drug safety. R&D projects to address the issues presented in this study will have to be planned in the future. For that process, it will be helpful to perform a scenario analysis. The scenario would provide a direction for the allocation of resources for problem resolution and enable the efficient use of limited resources. Continuous and periodic identification of trends and issues in drug safety will be required to determine policies proposed by government agencies for drug safety management and the direction of R&D to be carried out.

## 5. Conclusions

Twenty key issues related to drug safety were derived using scientific foresight methodologies. ‘Illegal distribution of medicines’ and ‘Lack of technology for managing and utilizing big data’ were identified as the most important key issues. The key issues could be used to establish plans for medication safety management. To address the issues, MFDS will need to plan its R&D strategy. This will require further research such as SWOT (Strength, Weakness, Opportunity, and Threat) analysis and scenario analysis.

## Figures and Tables

**Table 1:** Ten trends in safety management of medicines.

Trends in Drug Safety
1. Application of the 4th Industrial Revolution and artificial intelligence (AI) in the field of medicines
2. Drug safety for the aged, pregnant women, and multicultural families
3. The international harmony of regulatory science
4. Introduction of illegal medicines due to increased foreign trade
5. Introduction of precision medicines
6. Preparing for terrorism and disaster
7. Communication of medication safety information with the public.
8. Encourage generic drug use
9. Novel variables for medication efficacy and safety assessment
10. Drug safety in preparation for the unification of Korea

**Table 2:** Urgency and impact of issues in safety management of medicines.

Issues	Urgency	Impact	Average
Key issues (total score ≥ 4)			
Illegal distribution of medicines	5.96	5.08	5.52
Lack of technology for managing and utilizing big data	5.11	5.84	5.48
Change in the pharmaceutical trade environment	5.59	5.08	5.34
Lack of education and safety management for specific populations (pediatrics, elderly, etc.)	4.90	5.46	5.18
Lack of artificial intelligence-based technology for safety management of medicines	4.73	5.54	5.14
Prevalence of drug advertisements through social network services	5.04	5.15	5.10
The necessity of novel parameters and models for drug efficacy and safety measurement	4.36	5.39	4.88
Changes in drug effectiveness and safety in specific populations	4.09	5.61	4.85
National factors of multiregional clinical trials	5.01	4.69	4.85
Securing equivalence of generic drugs	5.21	4.31	4.76
Needs for real-time medication quality management	4.35	5.08	4.71
Needs to implement international drug regulations	4.39	4.85	4.62
Lack of infrastructure for the introduction of precision medicines	3.47	5.46	4.47
Responsibility for leading new regulatory directions	4.32	4.54	4.43
Development of medications applied with new convergence technology with insufficient safety management regulations	4.24	4.30	4.27
Lack of evaluation of fertility and maternity drugs	3.63	4.77	4.20
Need for rapid analysis and evaluation of new illegal drugs	4.67	3.54	4.11
Lack of communication skills for delivering medicines information	4.24	3.84	4.04
A shortage of medicines in the event of terrorism and disasters	3.78	4.30	4.04
Technical gaps in safety management of medicines between South and North Korea	3.50	4.50	4.00
Issues other than key issues (total score < 4)			
Novel genotype for drug efficacy and safety	2.90	4.85	3.88
Delay in introducing medical countermeasures	2.92	4.38	3.65
Difficulties in preparing children’s medicines	3.01	3.75	3.38
Inconsistency of the medication management system between South and North Korea	2.32	2.61	2.47

## Supplementary Materials

The following are available online at https://www.mdpi.com/1660-4601/16/18/3368/s1, Table S1: Reference future reports; Table S2: Global megatrends.
