# Supplementary material for: Analysis of Issues and Future Trends Impacting Drug Safety in South Korea
Source: Int J Environ Res Public Health. 2019 Sep 12;16(18):3368. doi: 10.3390/ijerph16183368 (PMC6765849; doi:10.3390/ijerph16183368)
Supplement: Supplementary file 1 [file ijerph-16-03368-s001.pdf]

Future Trends and Issues in Drug Safety Areas in South Korea

Supplementary

Table 1. Reference future reports

Title	Authors	Year of publication
Production and Consumption in the Fourth Industrial Revolution of Korea 10 years later: Future Issue Reports [8]	Ministry of Science, ICT and Future Planning, KISTEP, KAIST	2017
The Global Risks Report 2017 [9]	World Economic Forum	2017
2045 World Future Report [10]	The Millennium Project	2016
Korea in 10 Years: Future Issue Reports [11]	Ministry of Science, ICT and Future Planning, KISTEP, KAIST	2015
Investigation on Long-term Challenges for Future Strategies [12]	KISTEP	2015
Outlook on the Global Agenda 2015 [13]	World Economic Forum	2014
The four global forces breaking all the trends [14]	McKinsey Global Institute	2015
2015-16 State of the Future [15]	The Millennium Project	2015
Future State 2030: The global megatrends shaping governments [16]	KPMG International	2014
Global Strategic Trends - Out to 2045 [17]	Ministry of Defence of United Kingdom	2014
A Survey on the Demand for Future Radio Technologies in 2025 [18]	National Radio Research Agency	2013
Technology Forecasting 2040: Land, Infrastructure and Transport [19]	Korea Agency for Infrastructure Technology Advancement	2013
Foresight Future Identities [20]	The Government Office for Science	2013
"Future vision 2050 " A great society where the people are happy [21]	Korea National Council on Social Welfare	2012
The Future of ICT in 2025 [22]	KEIT	2012
Global Trends 2030: Alternative Worlds [23]	National Intelligence Council	2012
Megachange - the World in 2050 [24]	The Economist	2012
25 Things You Need To Know About The Future [25]	Barnatt C	2012
Global Europe 2050 [26]	European Commission	2012
Germany 2030: Future perspectives for value creation [27]	Federation of German Industry (BDI), Z_punkt	2012
The 4th Science and Technology Foresight (2012~2035) [28]	KISTEP	2012
Das Megatrend-Prinzip: Wie die Welt von morgen entsteht [29]	Horx M	2011
IT Convergence Future Technology Forecast Survey 2025 [30]	KEIT	2011
(The) Change [31]	Samsung Economic Research Institute	2011

2020 A new future is coming [32]	LG Economic Research Institute	2010
10 Years of Global Trend [33]	Trends Magazine	2010

ICT = Information and Communication Technology, KAIST = Korea Advanced Institute of Science and Technology, KEIT = Korea Evaluation Institute of Industrial Technology, KISTEP = Korea Institute of Science and Technology Evaluation and Planning

Table 2. Global megatrends

STEEP	Global megatrends	Keywords
Social (S)	1. Structural and functional changes of social members	Aging, low birth rate, increasing health risks, generation change, breaking up the existing family system, increasing female status, and increasing gender issues
	2. Deepening social polarization	Widening income/wealth gap, potential social unrest due to income inequality, the polarization of industrial structure, and urbanization
	3. Proliferation of education and consumerism	The spread of education, increase of authority through education, strengthen the knowledge-based economy, change in consumer requirements, and consumerism
	4. Increasing awareness of the quality of life	Changing patterns of cultural consumption and enjoyment, increasing the value of leisure and cultural activities, establishing a living place for health promotion, and a lifestyle that emphasizes the quality of life
	5. Disaster and cyber crime	Social disasters, man-made disaster (oil leaks, nuclear accidents, etc.), cyber-attack/crime, hacking, massive data fraud/theft, and digital nomad
	6. Globalization	Spread of multicultural society, global connection, and living with foreigners
	7. Increasing demand for social security system	Creating a future infrastructure, emerging new social services models, breaking the boundaries of public and private sectors, and ensuring larger infrastructure
Technological (T)	8. Bio-technology and life extension	Biotechnology, stem cells, genetic modification, man-made life, genetic medicine, lab on a chip, instant disease diagnosis, vaccine revolution, and cancer prevention
	9. Cognitive science	Reinforcement of cybernetic, expansion of cognitive science, nerve technology, and human enrichment
	10. Space engineering	Space age, the discovery of extraterrestrial life, space travel, and space development
	11. Information and communication technology, robot, and artificial intelligence	Ubiquitous connection sensors, hyper-connected society and big data utilization, internet of things, wearable devices, cloud computing, mobile communication, advanced search engine, social network proliferation, virtual and augmented reality, robot and artificial intelligence development, and process automation
	12. New material technology	New materials, advances in nanotechnology, bio-printing, advances in 3D printing, dimensional printing
	13. Transportation technology	Advanced transport technology, sustainable transportation, high-speed transportation, logistics chaos, global transportation congestion, and new intelligent logistics concept
	14. Inter-disciplinary fusion	Accelerating technology convergence, creating through fusion, synthetic biology, biotechnology and information technology convergence, and micro-robots in the body
Environmental (E)	15. Environmental change and disaster	Climate change and natural disaster, environmental destruction, biodiversity loss and ecosystem collapse, food shortages, green technology, increased threat of new diseases, and pandemic disease
	16. A scarcity of resources	Energy engineering technology (production, storage, transmission), resource depletion, use of alternative energy (such as renewable energy), resource protection, and importance of sustainability

Economical (E)	17. Low growth in major countries	Asset bubbles and deflation in major countries, major financial mechanisms and institutions failure, high structural unemployment and incomplete employment, prolonged era of low growth, rising national debt, and decreasing the ability to deal with future global economic and financial crises
	18. Emerging countries	Rise of developing countries, development of Asia and Middle East, population growth in Africa, and increased Korea's global status
	19. Emergence of a new global economic system	Sharing economy, economic democratization, digital currency, economic globalization, increasing importance of international partnerships, financial regulations and global financial safety net
	20. Protectionism	The emergence of protectionism and foreign policy for realizing national interests
	21. Paradigm changes in manufacturing and supply	Personalization of supply, pattern changes in markets, change of manufacturing paradigm, revolution in manufacturing, new future of manufacturing, digital convergence of processes, niche strategies, online transactions
Political (P)	22. International conflict	Weakness of international governance, conflicts between nations, large-scale terrorist attacks, increasing international organized crime, ideological conflict with neighboring countries, and refugees
	23. Unification problem	Unification issues rising, inter-Korean exchange and unification problems
	24. Democratic development	Electronic democracy, social network politic, network-centric power shift, new civil society, the maturity of democracy, greater freedom of speech and expression
	25. Reliability and transparency of political administration	Corruption, ethical considerations in decision making, governance failures, public pension needs, public debt growth, and government administration efficiency
